# Prognosis and Risk Factors of Coronary Artery Lesions before Immunoglobulin Therapy in Children with Kawasaki Disease

**DOI:** 10.4274/balkanmedj.galenos.2020.2020.1.56

**Published:** 2020-10-23

**Authors:** Huixian Qiu, Chang Jia, Zhenquan Wang, Yuee He, Xing Rong, Rongzhou Wu, Maoping Chu, Hongying Shi

**Affiliations:** 1 Children’s Heart Center, The Second Affiliated Hospital & Yuying Children’s Hospital, Institute of Cardiovascular Development and Translational Medicine, Wenzhou Medical University, Zhejiang, China; 2 Department of Preventive Medicine, School of Public Health and Management, Wenzhou Medical University, Zhejiang, China; # These authors contributed equally to this work

**Keywords:** Coronary artery lesions, Kawasaki disease, prognosis study, survival analysis

## Abstract

**Background:**

Many children with Kawasaki disease develop coronary artery lesions before intravenous immunoglobulin treatment. However, little data are available on the prognosis of children with Kawasaki disease who developed coronary artery lesions before intravenous immunoglobulin treatment.

**Aims:**

To explore the outcomes of coronary artery lesions before intravenous immunoglobulin treatment in children with Kawasaki disease and analyze the factors that influence the duration of coronary artery lesions.

**Study Design:**

Retrospective cohort study.

**Methods:**

All patients with Kawasaki disease who developed coronary artery lesions before intravenous immunoglobulin treatment in our hospital from January 2009 to December 2014 were reviewed. A Cox proportional hazards model was used to determine the factors influencing the prognosis of coronary artery lesions.

**Results:**

Among 182 patients included, 28.6% were male, 83.50% were younger than 36 months, and 181 exhibited resolution of coronary artery lesions 2 years after disease onset. The median duration of coronary artery lesions was 31 days, and the proportion of coronary artery lesions was 52% at 1 month, 35% at 2 months, 33% at 3 months, 25% at 6 months, 14% at 1 year, and 0.5% at 2 years. The univariate analysis showed that overweight status, higher platelet count, lower albumin level, and starting treatment more than 10 days after disease onset were factors that possibly affect the duration of coronary artery lesions in children. The multivariate Cox regression analysis showed that female sex (adjusted hazard ratio, 1.661; 95% confidence interval, 1.117-2.470) was an independent protective factor, and overweight status (adjusted hazard ratio, 0.469; 95% confidence interval, 0.298-0.737), higher platelet count (adjusted hazard ratio, 0.649; 95% confidence interval, 0.443-0.950), and starting treatment more than 10 days after disease onset (adjusted hazard ratio, 0.392; 95% confidence interval, 0.215-0.716) were independent risk factors for a longer duration of coronary artery lesions.

**Conclusion:**

The average duration of coronary artery lesions before intravenous immunoglobulin therapy in children with Kawasaki disease is approximately 1 month. Male gender, overweight status, higher platelet count, and initiation of treatment more than 10 days after the onset of the disease are independent risk factors for longer-lasting coronary artery lesions.

Kawasaki disease (KD) is often complicated by coronary artery damage (1,2). Coronary artery lesions (CAL) affect the prognosis of children with KD and occur in 25% to 30% of children with untreated KD (1,3,4). Cardiac prognosis is significantly improved with treatment of 2 g/kg of human intravenous immunoglobulin (IVIG) (5,6). A study in our department showed that the proportion of children with CAL in acute-stage was about 20% (7). Left ventricular strain is impaired in patients with KD at a median follow-up of 57.5 months (8); however, 99.8% of children with maximum z scores of less than 2.5 at ≤10 days or 2 weeks would return to normal by the 6th week after illness onset (9). We found that many children developed CAL before IVIG treatment. However, little data are available on the prognosis of patients with KD who developed CAL before IVIG treatment, including the duration of CAL, what proportion of CAL will disappear, and the factors that influence the prognosis of CAL.

In this study, patients with KD who developed CAL before IVIG treatment were included. The duration of CAL, and the factors that influence the duration of CAL were analyzed.

## MATERIALS AND METHODS

### Subjects

We collected the medical records of all KD inpatients at our hospital, from January 1, 2009 to December 31, 2014. Patients who developed CAL before IVIG treatment were enrolled, and those who did not received IVIG treatment or did not have echocardiography findings both during hospitalization and after discharge were excluded.

All patients were treated with IVIG at a dose of 2 g/kg, and aspirin at a dose of 30-50 mg/kg per day. For IVIG-resistant patients, an additional IVIG dose of 2 g/kg was administered. All patients were followed up to disappearance of CAL, or 2 years after treatment.

The diagnosis of complete KD is based on the presence of fever for 5 days or more, and at least four of the following five symptoms (10): bilateral conjunctival injection without exudates, oral change, extremity changes, polymorphous rash, and/or cervical lymphadenopathy. Patients with fever for 5 days or more and at least two of the principal features can be diagnosed with incomplete KD, if no other disease process explains the illness.

The patients underwent echocardiographic examination at diagnosis (before IVIG treatment) and at 1 month ±7 days after disease onset, as well as 2 months ±7 days, 6 months ±2 weeks, 1 year ±1 month, and 2 years ±1 month after disease onset. We collected the absolute dimensions of the proximal right coronary artery, left main coronary artery, and left anterior descending artery.

The diagnosis of CAL was based on the following three criteria: (1) Coronary artery diameter of >2.5 mm in children younger than 3 years old; >3.0 mm in children 3 to 9 years old; and >3.5 mm in children older than 9 years old, as well as the diameter of one segment of the coronary artery more than 1.5 times that of the adjacent segment; (2) coronary artery aneurysm: the ratio of the diameter of the coronary artery to the adjacent segment is >1.5, and the diameter of the coronary artery is >4 mm; (3) coronary artery stenosis and embolism.

### Methods

We collected the following information: age at onset, gender, and body mass index (BMI). We collected clinical characteristics including the time of diagnosis, time of initial IVIG treatment, type of KD, and resistance to IVIG treatment. Additionally, any changes in the medical department were recorded. We also collected laboratory data including the platelet count, C-reactive protein (CRP) level, hemoglobin level, albumin level, and alanine transaminase level at baseline.

This study was approved by the ethical Board of The Second Affiliated Hospital & Children’s Hospital of Wenzhou Medical University, Zhejiang, China (LCKY2019-16). Requirement for individual consent was waived for this retrospective study.

### Statistical methods

Quantitative variables are presented as mean ± standard deviation if they were normally distributed, and presented as median [interquartile range (IQR)] if they were not normally distributed. Qualitative variables are presented as frequency (percentage). CAL regression curves were drawn by the Kaplan-Meier method, and the differences in CAL regression between different groups were compared using the log rank test. The crude and adjusted hazard ratios (HRs) and their 95% confidence intervals (CIs) were estimated by a Cox proportional hazards regression model to estimate the risk factors for prognosis of CAL before IVIG treatment in patients with KD. Data were analyzed with SPSS software version 23.0 (IBM Corp., Armonk, NY, USA) and EmpowerStats (www.empowerstats.com; X&Y Solutions Inc.). All tests were two-sided and considered significant at the 0.05 level.

## RESULTS

### Basic characteristics

Among 182 patients included in the study, 28.6% were male and 83.50% were younger than 36 months. The mean BMI was 17.80±2.78 kg/m^2^, and the proportion of overweight patients was 24.5%. Incomplete KD was present in 53.8% of patients. The CRP level was >150 mg/L in 18.1% of patients, and the hemoglobin level was <100 g/L in 19.2% of patients. The median time of diagnosis was 5 days (IQR, 4-7 days), and the median time of receiving IVIG treatment was 7 days (IQR, 6-9 days). Only nine patients (4.9%) were resistant to IVIG treatment (Table 1). No patients died during the follow-up period.

### Outcomes of patients with CAL before IVIG treatment

**Figure 1:**
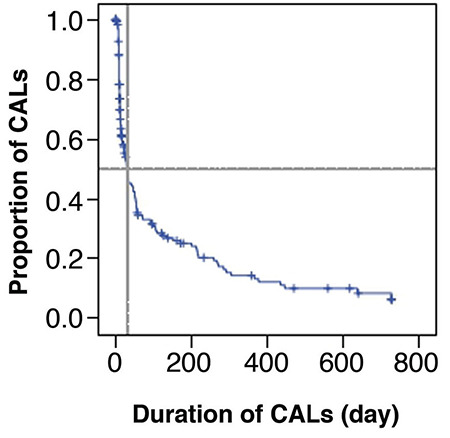
Proportion of coronary artery lesions at different time points. CALs: coronary artery lesions

The median duration of follow-up was 1 month (IQR, 1-3 months). The CAL of 181 patients ultimately disappeared, and the median time of recovery was 31.0 days (IQR, 17.8-44.2 days). In total, 52% of patients still had CAL at 1 month. The proportion of CAL at 2 months, 3 months, 6 months, and 1 year after illness onset was 35%, 33%, 25%, and 14%, respectively; by 2 years after onset, only 0.5% of patients still had CAL (Figure 1).

### Factors affecting the duration of CAL (log rank test)

**Figure 2:**
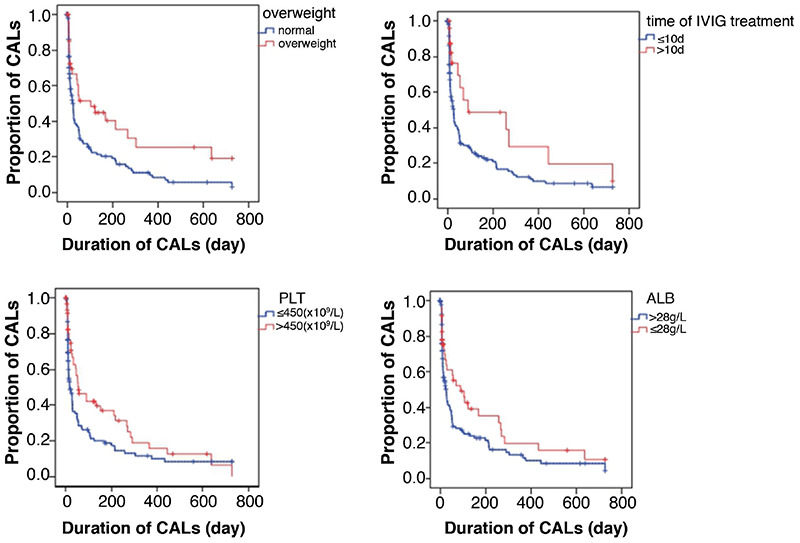
Factors that affected the duration of coronary artery lesions. CALs: coronary artery lesions

We then analyzed whether overweight status was associated with the duration of CAL. The results showed a higher proportion of CAL among overweight children than children of normal weight at the same time point (*X*^2^=8,285, p=0.004). We also found that patients who started IVIG treatment more than 10 days after onset required a longer time to recover to a normal clinical condition than those who started IVIG treatment within 10 days (*X*^2^2=5.702, p=0.017). Patients with a lower albumin level (<28 g/L) and higher platelet count (>450×10^9^/L) had a longer duration of CAL than patients with an albumin level of ≥28 g/L and a platelet count of ≤450×10^9^/L (Figure 2). However, we found no evidence that age, sex, type of KD, change in medical departments, or hemoglobin level was associated with the duration of CAL in univariate analysis.

### Factors affecting resolution of CAL before IVIG treatment (Cox regression

The results from Cox regression model indicated that female sex was an independent protective factor (adjusted HR, 1.661; 95% CI, 1.117-2.470). Overweight status (adjusted HR, 0.469; 95% CI, 0.298-0.737), high platelet count (adjusted HR, 0.649; 95% CI, 0.443-0.950), and starting IVIG treatment more than 10 days after onset (adjusted HR, 0.392; 95% CI, 0.215-0.716) were independent risk factors for resolution of CAL (Table 2).

## DISCUSSION

In this study, we identified 930 patients with KD, 182 (19.6%) of whom developed CAL before IVIG treatment. The proportion of patients with CAL before IVIG treatment was lower than previously reported. De Ferranti et al. (9) found that about 31.6% (318/1203) of patients had CAL at baseline (within 10 days of onset), most children were younger than 3 years old, and the proportion of male children was 71.4%. In our study, the proportion of children younger than 36 months old was 83.50%, and the proportion of male children was 28.6%. Downie et al. (10) reported that male children with delayed or no treatment were more likely to develop coronary artery aneurysms. In recent years, studies have shown that 5.0% to 38.3% of patients with KD are resistant to IVIG (11,12,13). In the present study, we found that 4.9% patients were resistant to IVIG treatment; this proportion is lower than in previous studies. During a follow-up of 2 years, the CAL of 181 (181/182) patients ultimately disappeared, and the median time of recovery was 31.0 days.

Chbeir et al. (14) reported that about 31% of patients presented with echocardiographic abnormalities, which were strongly associated with resistance to IVIG and development of CAL within the first 6 weeks of disease; however, they did not focus on the factors that influence the prognosis of CAL. We analyzed the prognosis of CAL before treatment and investigated the factors that possibly affect the duration of CAL. BMI is a risk factor for cardiovascular events. Excessive BMI increases cardiovascular mortality in adults and is associated with further cardiovascular events in patients with KD (15,16). Therefore, we analyzed the effect of weight on the prognosis of CAL, and the results indicated that overweight children had a higher proportion of CAL than children of normal weight at the same time point. Additionally, overweight children required a longer time to return to a normal clinical condition, which is consistent with our previous study (7,17). Platelet count has been used in a risk scoring system to predict IVIG resistance and coronary artery abnormalities (18,19,20). Bozlu et al. (21) showed that children with KD have lower mean platelet volume-tolymphocyte ratio compared with control subjects. In this study, we found that the duration of CAL was longer in children with a higher platelet count, and a platelet count of >450×10^9^/L was an independent risk factor for prognosis of CAL. Previous studies have shown that a low albumin level is associated with CALs (22,23). We found that the proportion of CAL was higher in children with a low albumin level than in those with an albumin level of >28 g/L, indicating that children with a low albumin level need more time to attain a normal clinical condition. Children with KD should receive IVIG treatment within 10 days of illness onset, and treatment within 7 days of onset is optimal (24,25). We found that the proportion of patients with incomplete KD was higher than that of patients with complete KD. Because of the lack of clinical manifestations in children with incomplete KD, they always undergo delayed diagnosis and treatment; thus, delayed IVIG treatment is associated with a higher risk of developing CAL during the convalescent phase (7,16,26). We also found that the duration of CAL was longer in children with delayed IVIG treatment.

Approximately 25% patients with KD develop serious coronary artery abnormalities, such as coronary artery aneurysm and ectasia, if left untreated (27,28). The patient’s age, duration of symptoms, prior hospitalization, and platelet count have been identified as independent predictors of coronary artery abnormalities (20). In one study, predictors of IVIG resistance and coronary artery abnormalities in patients with KD were aspartase transaminase level of ≥100 IU/L, sodium level of ≤133 mmol/L, duration of illness before initial treatment of ≤4 days, neutrophil level of ≥80%, CRP level of ≥10 mg/dL, age of ≤12 months, and platelet count of ≤30.0×10^4^/mm^3^ (19). We also analyzed the factors that may affect the duration of CAL, and the results indicated that female sex was a protective factor for the prognosis of CAL, while overweight status, higher platelet count, lower albumin level and delayed IVIG treatment were independent risk factors for the prognosis of CAL. These findings are consistent with a previous report (9). However, the levels of alanine transaminase and hemoglobin had no association with the duration of CAL. Additionally, the type of KD had no effect on the duration of CAL.

This study had several limitations. Firstly, this is a retrospective study, and all patients were from a single Pediatric Cardiac Clinic, which might have caused selection bias. Secondly, in our center, conventional or CT angiography is not performed routinely, therefore, comparison between echocardiography results and conventional or CT angiography findings cannot be made. Finally, we did not use z score for assessment of CAL.

Many children with KD developed CAL before IVIG treatment, and the duration of CAL was longer in children with overweight status, low albumin level, high platelet count, and treatment later than 10 days after onset. Female sex was a protective factor for the prognosis of CAL, while overweight status, high platelet count, and delayed IVIG treatment were independent risk factors for the prognosis of CAL.

## Figures and Tables

**Table 1:**
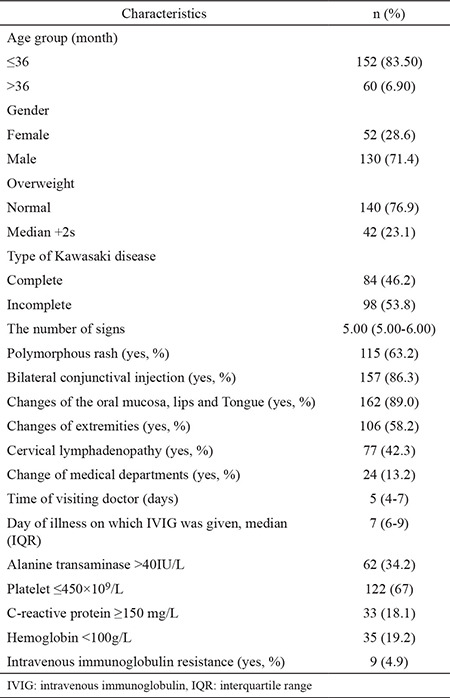
Baseline characteristics of patients who developed coronary artery lesions before intravenous immunoglobulin treatment

**Table 2:**
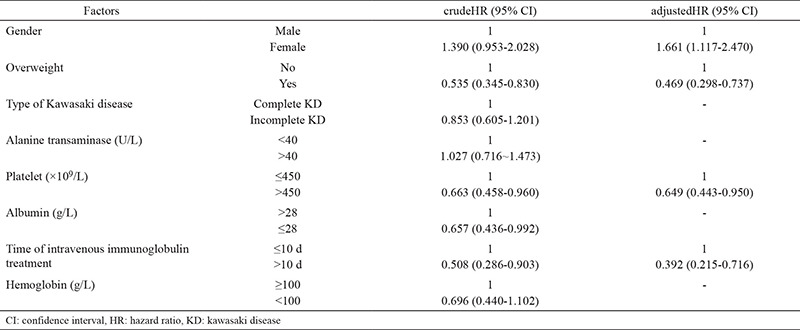
Factors associated with resolution of coronary artery lesions before intravenous immunoglobulin treatment
